# Computational analysis of whole slide images predicts PD-L1 expression and progression-free survival in immunotherapy-treated non-small cell lung cancer patients

**DOI:** 10.1186/s12967-025-06487-2

**Published:** 2025-05-06

**Authors:** Abdou Khadir Dia, Alona Kolnohuz, Sevinj Yolchuyeva, Marion Tonneau, Fabien Lamaze, Michele Orain, Andréanne Gagné, Florence Blais, François Coulombe, Julie Malo, Wiam Belkaid, Arielle Elkrief, Drew Williamson, Bertrand Routy, Philippe Joubert, Mathieu Laplante, Steve Bilodeau, Venkata SK. Manem

**Affiliations:** 1 Centre de Recherche du CHU de Québec - Université Laval, Québec, Canada; 2 Quebec Heart & Lung Institute Research Center, Québec, Canada; 3 Centre de Recherche du Centre Hospitalier Universitaire de Montréal, Montréal, Canada; 4 Department of Molecular Biology, Medical Biochemistry and Pathology, Université Laval, Québec, Canada; 5 Université de médecine de Lille, Lille, France; 6 Department of Pathology and Laboratory Medicine, Emory University School of Medicine, Atlanta, USA; 7 Université Laval, Québec, Canada; 8 Cancer Research Center, Université Laval, Québec, Canada; 9 Big Data Research Center, Université Laval, Québec, Canada

## Abstract

**Background:**

Immune checkpoint inhibitors (ICIs) have revolutionized cancer treatment by significantly improving the efficacy of treatments and tolerability for patients with non-small cell lung cancer (NSCLC). However, even after meticulous selection based on molecular criteria, only 20–30% of the patients respond to ICIs. This highlights the urgent clinical need to develop more precise biomarkers to better identify individuals who will benefit from these expensive therapies.

**Methods:**

Data from NSCLC patients treated with immunotherapy were collected from two institutions. From the histological images of tumors, pathomics features were extracted. We employed six machine learning models and seven feature selection methods to predict expression of the programmed death-ligand 1 (PD-L1), a current biomarker used to select patients for immunotherapy, and progression-free survival (PFS). The association between pathomics features and biological pathways was explored to validate pathomics-based signatures. We performed gene set enrichment analysis to identify the pathways enriched with the predictive signatures.

**Results:**

Handcrafted histological features were extracted from the whole slide images (WSI). The Support Vector Machines model with the SurfStar feature selection method, offered the best results, with an area under the curve (AUC) of around 0.66 for both the training and validation sets to predict PD-L1. For the prediction of PFS, the most effective model was linear discriminant analysis using the Multi Surf feature selection method with an AUC of 0.71 for the training set and 0.62 for the validation set. We found immune pathways to be upregulated in the high PD-L1 and high PFS groups, confirming the utility of image analysis for predicting clinical endpoints in patients treated with immunotherapy.

**Conclusion:**

Our models, based on the analysis of histological images, can serve as predictive biomarkers for PD-L1 and PFS. This approach, focused on histological images, enables the distinction of patients based on treatment response, thus providing clinicians with a valuable tool for patient management. With further validation on external cohorts, these models could enhance clinical decision-making through analysis of routine medical images.

## Introduction

Traditional systemic therapies have long been the cornerstone of treatment for advanced non-small cell lung cancer (NSCLC) patients; however, their effectiveness has now plateaued. Therapeutic options for NSCLC are limited and associated with low response rates and significant toxicities [1], largely due to the late diagnoses. However, the advent of immune checkpoint inhibitors (ICIs) has marked a turning point, significantly improving treatment efficacy and tolerability for patients presented with NSCLC, compared to traditional therapeutic interventions [2]. Programmed death-ligand 1 (PD-L1) expression is currently the only validated biomarker for guiding the use of ICIs, influencing treatment choice based on the disease stage and the type of ICI. Although systemic treatments have long been the standard of care for advanced NSCLC, the emergence of ICIs has profoundly changed clinical paradigm and therapeutic options for these patients, surpassing the results obtained with platinum-based chemotherapies [3]. Research is now directed towards innovative combinations of ICIs with other treatments to overcome therapeutic failures and improve personalized outcomes [4]. Nonetheless, primary and secondary resistance to ICIs presents a major challenge, necessitating the development of predictive biomarkers for more accurate patient selection who are likely to benefit from these treatments.

In this complex context, ICIs have established a new standard of care for advanced NSCLC, offering survival prospects previously unattainable. However, the variable response to ICIs, even among patients selected based on PD-L1 expression, underscores the urgent need to identify more reliable predictive biomarkers. The goal is to avoid unnecessary toxicities and maximize the chances of response by targeting treatments to the patients most likely to benefit, highlighting the crucial importance of ongoing research to refine and personalize therapeutic strategies against NSCLC.

With the emergence of digital pathology, the generation of data in the form of high-resolution images during tumor biopsy diagnostics has become common in clinical settings. These images, now indispensable tools, are utilized by researchers to develop predictive and prognostic biomarkers leveraging machine learning and deep learning models. These models, particularly those based on deep learning, have proven effective for detecting, classifying certain tumors and predicting treatment responses [5]. For example, Vanguri et al. [5] developed a dynamic deep attention-based multiple-instance learning model with masking (DyAM) from genomic, histological, and radiological data to predict the response to immunotherapies, using RECIST criteria as an indicator of response in NSCLC patients treated with PD-L1 inhibition. To generalize the results, it is necessary to validate the model on a larger and multimodal cohort. Other studies using deep learning to predict the response to immunotherapy have been conducted, although their lack of interpretability limits their clinical use. To develop both efficient and interpretable models, research efforts have been undertaken to employ machine learning methods. In this regard, Wang et al. [6] utilized tumor-infiltrating lymphocytes (TIL) in the tumor microenvironment to predict the response to ICIs by extracting quantitative histomorphometric features from histological images. Despite validation on various cohorts, these models require further biological validation and training on larger datasets. Ding et al. [7] developed machine learning models using pathomic features to differentiate morphological and molecular aspects of immune responses in Hematoxylin and Eosin (H&E) images, though an automatic selection of patches may introduce biases. Although several models for predicting treatment response and survival have been developed, they exhibit significant limitations. The variability in radiological images, due to differences in acquisition parameters or the type of equipment used, compromise model performance. Furthermore, models are often trained and validated on the same cohort and suffer from generalization problems and overfitting, especially due to the large number of features extracted from pathological or radiological images. The lack of biological validation also poses a major hurdle affecting the reliability of predictions regarding survival or treatment response.

Developing prognostic and predictive models is a complex process, as no single modeling approach outperforms others. Additionally, validating these models necessitates the use of multi-institutional cohorts to demonstrate their applicability across various external datasets. Moreover, biological and external validation of these models require multi-institutional cohorts to demonstrate that the developed signatures are applicable to external datasets. In this study, we performed a systematic comparison leveraging a compendium of machine learning and feature selection approaches to develop predictive models for survival, leveraging multi-institutional WSI profiles of NSCLC patients treated with immunotherapy. We developed and biologically validated pathomics signatures predicting survival end points, and also PD-L1 expression, an area that is still underexplored. We explored various feature selection techniques to develop predictive models and minimize the risk of overfitting. By examining the association of pathomic features with biological pathways through gene set enrichment analysis (GSEA), we assessed the effectiveness of pathomic models as well as the relevance of different variable selection techniques to enhance prediction.

## Materials & methods

### Description of cohorts

This study retrospectively analyzed data from two prominent lung cancer treatment facilities: the Quebec Heart and Lung Institute at Université Laval (IUCPQ-UL) and the University of Montreal Hospital Centre (CHUM). Ethical approval for this research was granted by the respective institutional review boards (approval number MP-10-2020-3397). Eligible patients had a confirmed diagnosis of NSCLC and were treated with one of three immune checkpoint inhibitors (Atezolizumab, Nivolumab, or Pembrolizumab). Samples were collected between 2015 and 2021, with tissue slides prepared from archival biopsies. Patients were required to provide informed consent for the use of their clinical and pathological data in research. The selection of patient samples was based on strict inclusion criteria to ensure the suitability and clinical relevance of the dataset for pathomics analysis. Only patients with archival H&E-stained histological slides were included, ensuring that high-quality images were available for computational analysis. Additionally, all selected patients had a confirmed diagnosis of advanced non-small cell lung cancer (NSCLC) at the time of inclusion, as verified by pathology reports. To maintain a homogeneous treatment background, only patients who had received immune checkpoint inhibitors (ICIs), specifically Atezolizumab, Nivolumab, or Pembrolizumab, were considered. Furthermore, patients were required to have documented PD-L1 immunohistochemistry (IHC) scores, which served as a reference for validating the predictive performance of the pathomic models. Finally, adequate clinical follow-up data, including progression-free survival (PFS) outcomes, was essential for model training and evaluation, ensuring that the study findings could be meaningfully interpreted in a clinical context.

The tissue samples utilized in this research were sourced from the Quebec Respiratory Health Network Tissue Bank (accessible at https://rsr-qc.ca/biobanque/), located at the IUCPQ-UL. H&E-stained slides were digitized using a NanoZoomer 2.0-HT slide scanner with a 20X objective lens. From the CHUM and IUCPQ cohorts, 43 and 25 samples, respectively, were selected based on the availability of histological data. In total, the combined dataset from these institutions encompasses 68 patients, all included in the subsequent analyses.

### PD-L1 assessment

PD-1, a protein found on the surface of activated T cells, acts to suppress T cell activity when it binds to its ligands, PD-L1 and PD-L2 [8]. This interaction is a critical immune checkpoint in regulating immune responses. The presence of PD-L1 on tumor cells, also known as the tumor proportion score (TPS), is determined through immunohistochemical staining using the 22C3 clone (pharmDx kit) on a Dako Autostainer, a standard procedure in patient care following a lung cancer diagnosis. PD-L1 expression levels are quantified by the TPS, which measures the percentage of tumor cells exhibiting positive membranous staining, ranging from 0 to 100%. For clinical relevance, tumors are categorized based on PD-L1 TPS thresholds: less than 1%, between 1 and 49%, and 50% or higher. In our study, we have consolidated the first two categories into a single group.

### PFS assessments

Progression-free survival (PFS) is measured from the initiation of therapy to the point of disease progression or any cause of death, whichever comes first [9]. This metric is increasingly recognized as a critical, often primary, endpoint in cancer clinical trials for solid tumors due to its practicality and clinical relevance [10]. PFS duration is counted in days or months from treatment commencement to disease progression, death, or last known follow-up whichever occurs first with the latter scenario being treated as a censored observation. In our research, we aimed to develop a predictive model that distinguishes between responders (PFS > 12 months) and non-responders (PFS < 12 months) in patients undergoing treatment with ICIs. For this purpose, we categorized the collected datasets from CHUM and IUCPQ into two groups based on PFS: those exceeding 12 months and those falling below this duration.

### RNA sequencing analysis pipeline

RNA extraction from snap frozen primary tumour biopsies and resections was conducted using a Qiagen RNA extraction kit using the standard protocol (Qiagen). Prior library construction, mRNA was enriched using a polyA kit from 1 ug of total RNA. 400u of mRNA was used as input for library construction with the TruSeq^®^ RNA Sample Preparation kit (Illumina) following the protocol recommendation. Libraries were sequenced on a HiSeq™ 2500 Sequencing System (Illumina) at a depth of 25 million paired-end reads per sample of length of 75 bp. Quality control was done with Fastqc (version 0.11.9) [11]. Base pair below a phred score of 30 and adapter were clip off with trimmomatic/picard. Transcript abundance in transcript per million (TPM) was estimated using Kallisto (version v0.50.0) [12]. Transcripts were mapped on the reference genome GRCh38 and annotated with the GENCODE release 34 (GRCh38.p13). Gene-level estimated counts were computed using tximport (version 1.0.3) and applied a gene-level offset to correct for changes associated with transcript length with the option *countsFromAbundance="lengthScaledTPM”*. Estimated gene counts (*lengthScaledTPM)* were used for subsequent analyses.

### Whole slide image (WSI) pre-processing

Pre-processing whole slide images (WSIs) is an essential initial step in cancer research involving artificial intelligence, as it ensures the accurate extraction of patches. Digitizing WSIs can introduce various anomalies, including noise and background interference, which can affect the quality of the images. To address these issues, we employed the ‘histolab 0.06’ [13] module in Python, a sophisticated tool for WSI pre-processing. Our methodology involved a series of precise steps to enhance the WSIs’ quality. Initially, we converted the images to grayscale, which simplifies the detection of anomalies. Next, we applied Otsu thresholding to segregate the tissue regions from the background clearly. This was followed by binary dilation, which helps in expanding the regions of interest. Additionally, we implemented techniques (such as a chain of filters to calculate the tissue area mask) to eliminate small holes and remove insignificant objects from the images. This resulted in high-quality WSIs, predominantly containing tissue content of at least 80%. This rigorous pre-processing ensured that only the regions of interest, crucial for downstream analysis, were retained. Our primary objective was to develop a machine learning model that could predict PD-L1 expression and PFS using pathomic features and clinical data. Therefore, it was imperative to focus on those areas of the WSI rich in nuclei, as these are most relevant for our analysis.

The prioritization of regions abundant in nuclei within WSIs is driven by the objective of accurately representing the tissue’s cellular composition and pathological characteristics through the extracted patches. The pre-processing procedures were specifically developed to improve the visibility and contrast of these nuclei, thereby enabling precise identification and thorough analysis. This careful approach in pre-processing significantly helps in reducing any potential biases that might arise from non-tissue components or artifacts present in the WSIs. By focusing on nuclei-rich regions, our study enables a direct association between the features extracted from these regions and the clinical outcomes, particularly in terms of PFS and PD-L1 expression. This strategy aims to provide insights that are not only more interpretable but also highly relevant to clinical contexts. The meticulous selection of these regions for analysis is a crucial aspect of the study. It not only ensures the accuracy of the research but also strengthens the reliability of our conclusions about the relationship between pathomic features and important clinical endpoints like PFS and PD-L1. This approach is fundamental in understanding the complex interactions within pathomics and their implications in clinical outcomes.

### WSI segmentation and patching

The patches extracted from WSIs were of the dimensions 2000 × 2000 pixels, magnified 20X. We selected patches containing at least 15% nuclei coverage for extraction [14]. This extraction process utilized the ScoreTiler class from histolab (https://histolab.readthedocs.io/en/latest/readme.html), a tool designed to evaluate each patch, based on its nuclei count. The ScoreTiler function applies various methods including threshold-based techniques, color space conversion, watershed transformation, and morphological operations to enhance nuclei detection. These patches were then subdivided into tiles of 50 μm². Cell density estimation, a measure of the spatial arrangement of cells within a tissue, was performed on each patch as described by Alvarez-Jimenez et al. [15]. This involved segmenting nuclei and assigning a specific gray level to each patch, based on the estimated nuclei count [15, 16]. The output is a map visualizing cell density, highlighting the distribution of cells across different areas within the patch. Nucleus segmentation was achieved using the Watershed algorithm [17], after several preprocessing steps. These included removing small white noise and holes in the nuclei to define foreground and background regions. The nuclei areas were identified using distance transform and thresholding, followed by isolating non-nuclei areas through dilation. The Watershed algorithm [17] effectively delineates each nucleus by identifying peaks and valleys in the grayscale image, an essential step for dense regions. After segmentation, nuclei were classified based on size and shape, aiding in the analysis of cell density and distinguishing between different cell types and states.

### Extraction of pathomics features

Haralick features [18] represent a set of statistical metrics used to quantify the textures or patterns visible in an image. Originating from the gray-level co-occurrence matrix (GLCM), which tabulates the occurrences of specific pixel intensity value pairs based on their spatial relationship in an image, these features provide a numerical representation of texture. In the context of cell density maps, Haralick features facilitate the quantitative analysis of the spatial arrangement and patterns of cellular distribution. For each cell density map, these features were extracted by computing four distinct GLCMs, corresponding to horizontal, vertical, minor diagonal, and major diagonal orientations of pixel adjacency, as described in an earlier study [19].

### Feature selection methods

Haralick features facilitated the extraction of numerous variables from cell density maps. These continuous variables exhibit diverse distributions and correlations with the target outcome in our study. Since the goal is to develop machine learning models capable of accurately predicting PD-L1 expression and PFS, meticulous feature selection was carried out. Identifying the most relevant variables is critical for reducing redundancy and noise that could improve model interpretability, while minimizing the risk of overfitting.

To address the large number of features in the dataset, an initial data processing strategy was designed to narrow down the feature set to the most relevant variables. In this study, we implemented a two-step feature selection approach to develop predictive models for PD-L1 expression and progression-free survival. First, we computed the Spearman’s correlation coefficient and eliminated features that exhibited a correlation exceeding the threshold set at 0.9. 

Following this, five feature selection techniques were utilized to build the models [20]: analysis of variance (ANOVA) F-test (AFT), mutual information (MI), ReliefF (RL), Surf (SF), and Multisurf (MSF), as described below.

- The mutual information-based variable selection method assesses the amount of information gained about one variable by knowing the other. In other words, if two variables are independent, their mutual information is zero; conversely, if they are completely dependent, the mutual information is high. In selecting relevant variables, the mutual information of each explanatory variable is calculated in relation to the target variable. The variables deemed most pertinent for the predictive model are those that share the most information with the target variable [21].
- The ANOVA F-test method for variable selection evaluates the degree of linear dependence between two variables. It identifies significant variables by comparing their F-scores. Variables with the highest scores are considered to have a major impact on the dependent variable and are therefore often selected for inclusion in the final model [22].
- ReliefF is a feature selection method implemented in the Python package ‘scikit-rebate’ [23, 24]. This method, an extension of Relief, is optimized for better handling of data with missing features, multiclass classification problems, and complex datasets. ReliefF randomly selects examples from a dataset, identifies the nearest neighbors in the same and other classes, assesses the importance of variables based on their ability to differentiate these examples, and adjusts the variable scores with each iteration. These scores are accumulated over many repetitions to determine the most significant variables. Extensions of ReliefF, such as Surf, Multisurf, and SurfStar, automatically calculate the ideal number of neighbors to consider in the variable score evaluation.

### Pathomics model development

**Fig. 1:**
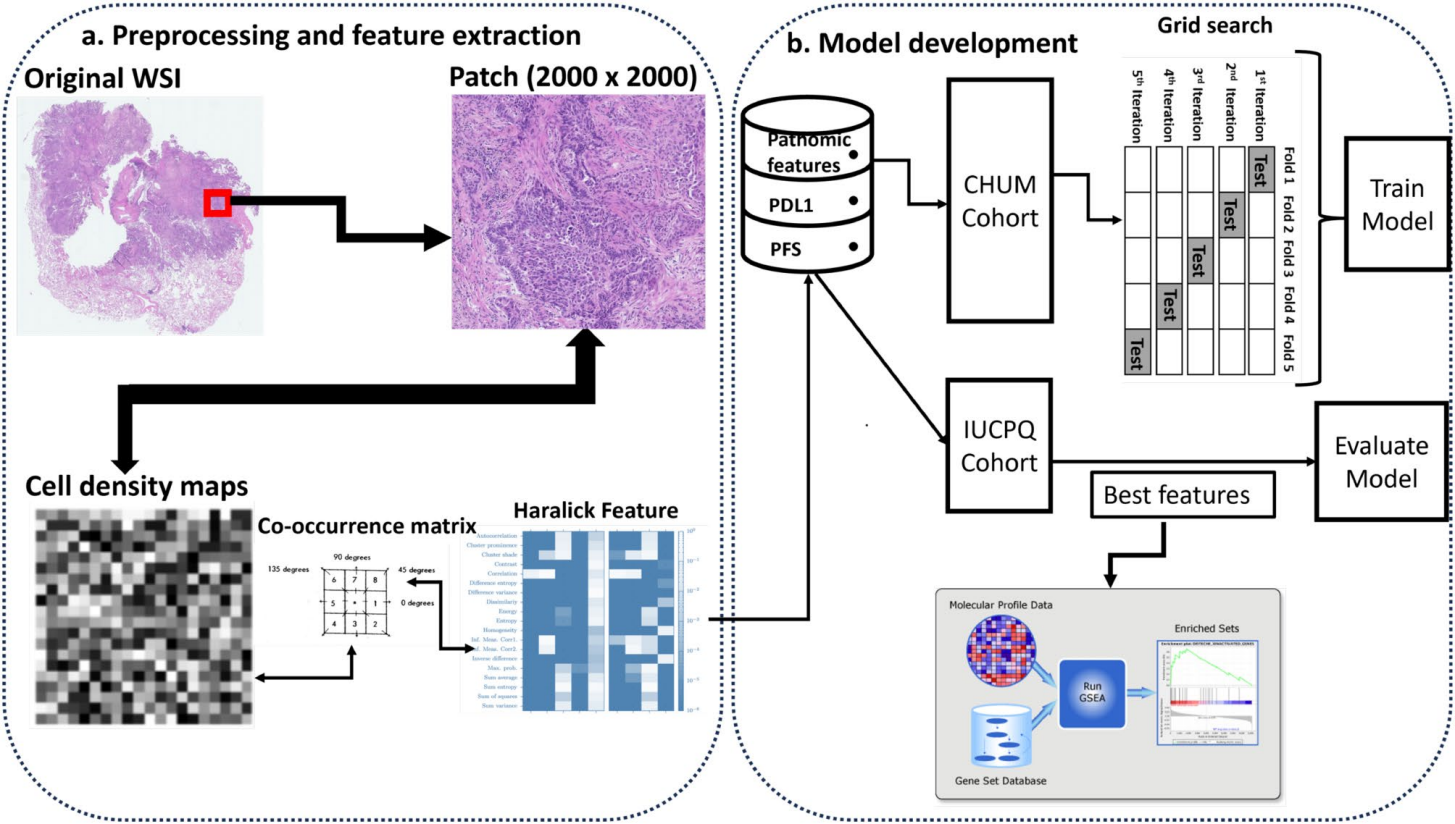
The workflow for the development of the model to predict PD-L1 expression and PFS from patients with NSCLC. **(A)** Whole Slide image pre-processing and feature extraction. **(B)** Model development and biological validation

The development of machine learning models involves several key steps. Data integrity is crucial for devising reliable models. In this study, we utilized histological images as input data, with PD-L1 expression and PFS as output variables. A meticulous process was applied to WSIs to ensure they were clean and free from defects, thus guaranteeing a tissue content of at least 80%. Following variable extraction, we normalized the data to achieve a mean of zero and a standard deviation of one, thereby standardizing the measurement units. We tested various machine learning models and feature selection methods to identify the optimal combination. The models examined include Adaptive Boosting (Adaboost), Decision Tree (DT), Random Forest (RF), Linear Discriminant Analysis (LDA), Support Vector Machines (SVM), K-Nearest Neighbors (KNN) and eXtreme Gradient Boosting (XG), all implemented via the Python scikit-learn package [25]. The analysis pipeline is illustrated in Fig. 1.

The model development phases include:

- Removing any empty rows among our variables of interest, resulting in 43 samples for the CHUM cohort and 25 for the IUCPQ-UL cohort. Due to data imbalance, we adopted the SVMSMOTE oversampling technique, a variant of the SMOTE algorithm, which uses SVM to identify samples suitable for generating new synthetic instances.
- Using the CHUM cohort as the training set, with validation on the IUCPQ cohort.
- Defining a training pipeline that includes feature selection, sampling method, and the model itself. For each model and at each iteration, we applied a variable selection method, a sampling technique, and then the model itself. To determine the optimal number of variables, we examined all available variables, choosing the number that provided the best AUC performance on the training and validation sets. This variable selection, carried out at each fold of the cross-validation, aims to prevent overfitting, especially critical given the small size of our samples.

### Biological validation of pathomics signature

To validate our model and confirm its biological relevance, we investigated the relationship between pathomics features and molecular pathways. The variables that contributed to the development of the most effective model were used to define a variable named “pathomic score”. This score was developed by initially calculating the median of the selected variables values for each patient. Subsequently, this variable was converted into two groups: high-risk group, if the variable’s value exceeds the median, and the low-risk group otherwise. Differentially Expressed Genes (DEGs) were identified between the high-risk and low-risk groups. The Gene Set Enrichment Analysis (GSEA) was conducted using the Kegg gene sets collection for GSEA, provided by the Molecular Signatures Database (MSigDB) [26].

### Pathway enrichment analysis

The pathway enrichment analysis was performed by employing the gene set enrichment analysis (GSEA) method using the statistic obtained from the t test (that assesses the difference in the gene expression between the two groups as defined in the previous section). The enrichment score for each pathway was computed using the GSEA method with statistical significance calculated using a permutation test (10,000 permutations). Nominal *P*-values obtained for each pathway was corrected for multiple testing using the false discovery rate (FDR) approach, and a threshold of *P* < 0.1 was considered statistically significant. The enrichment score is used as a fold change. If it is greater than 0 and FDR < 0.1, the gene is considered upregulated. Conversely, if it is less than 0 and FDR < 0.1, the gene is considered downregulated.

## Results

### Patient characteristics

Table 1 details the clinical characteristics of patients from the two cohorts used in this study. The CHUM cohort consists of 43 patients, of whom 55.8%, or 24 patients, have a PD-L1 level above 50%, and 26 patients have a PFS of less than 12 months, representing 60.5%. In the IUCPQ-UL cohort, 56% of the patients, or 14 individuals, have a PD-L1 below 50% and a PFS of less than 12 months. The average age in the CHUM cohort is 68 years with a standard deviation of 8, while in the IUCPQ_UL cohort, it is 66 years with a similar standard deviation. The CHUM cohort has a majority of women (58.1%) in contrast to the IUCPQ-UL cohort where women constitute 36% of the patients. It is noteworthy that all patients in both cohorts have a smoking history. In the CHUM cohort, 76.7% of patients are current smokers and 20.9% are former smokers, with only one patient, or 2.3%, never smoked. This trend is similar in the IUCPQ-UL cohort where 68% are current smokers, 24% are former smokers, and only 2 patients, or 8%, never smoked. The overall physical condition of the patients is assessed via the Eastern cooperative oncology group (ECOG) classification, indicating that 65% of patients in the CHUM cohort and 80% of the IUCPQ cohort have slightly restricted mobility but can perform light work. Some patients are self-sufficient but unable to work, active less often than the majority (25% in the CHUM cohort and 7.7% in the IUCPQ-UL cohort), while others spend most of their time at rest without working (9.4% in the CHUM cohort and 11.5% in the IUCPQ cohort).

**Table 1:** Clinical Characteristics of Discovery and Validation Cohorts

Clinical Features	PD-L1 and PFS	
	CHUM (Discovery)	IUCPQ (Validation)
No. of samples	43	25
Age, mean ± sd	68 ± 8	66 ± 8
Sex, n (%)		
Female	25 (58.1)	9 (36)
Male	18 (41.9)	16 (64)
Smoking Status, n (%)		
Former	9 (20.9)	6 (24)
Current	33 (76.7)	17 (68)
Never	1 (2.3)	2 (8)
ECOG status, n (%)		
1	21 (65.6)	21 (80.8)
2	8 (25)	2 (7.7)
3	3 (9.4)	3 (11.5)
PD-L1 expression, n (%)		
> 50%	19 (44.2)	11 (44)
< 50%	24 (55.8)	14 (56)
PFS, n (%)		
> 12 months	17 (39.5)	11 (44)
< 12 months	26 (60.5)	14 (56)

### Pathomics features

Whole slide image segmentation enabled the extraction of multiple patches per WSI, which were then converted into cell density maps. In our analysis, we worked with a set of 68 WSIs. Each WSI was subdivided into multiple image patches to facilitate processing and analysis. The total number of patches generated from the 68 WSIs is 11,157, which represents an average of 159 patches per WSI. This patch-based subdivision approach allows for better granularity in the analysis of image features while ensuring complete coverage of the information contained in each WSI. We extracted 13 Haralick features from four distinct gray-level co-occurrence matrices (GLCMs), based on cell density maps generated from each patch, thereby generating a total of 52 features per patch. Since PD-L1 expression is assessed across the entire WSI, it is consequently impossible to estimate the model at the patch level. Therefore, the model is estimated at the WSI level by calculating five statistics, such as the mean, median, variance, kurtosis, and skewness, for all patches and for each of the 52 features, resulting in a total of 260 features.

### Predictive efficacy of classification methods

**Fig. 2:**
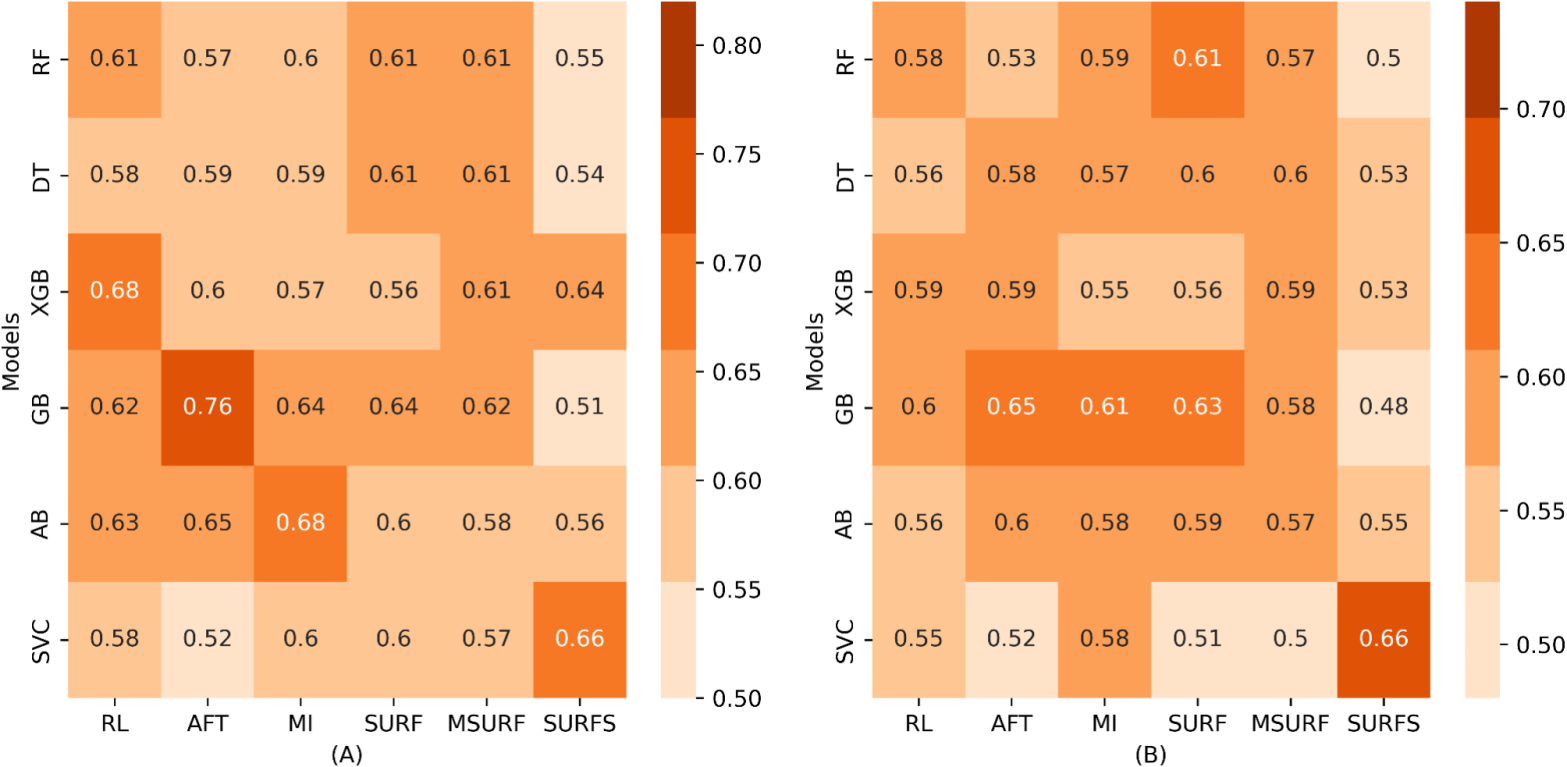
Heatmaps depicting the efficacy of various machine learning models (on the y-axis) across Different Feature Selection Techniques (on the x-axis) for Predicting PD-L1. **(A)** AUC Scores During Cross-Validation and **(B)** AUC Scores in the External Validation Phase

**Fig. 3:**
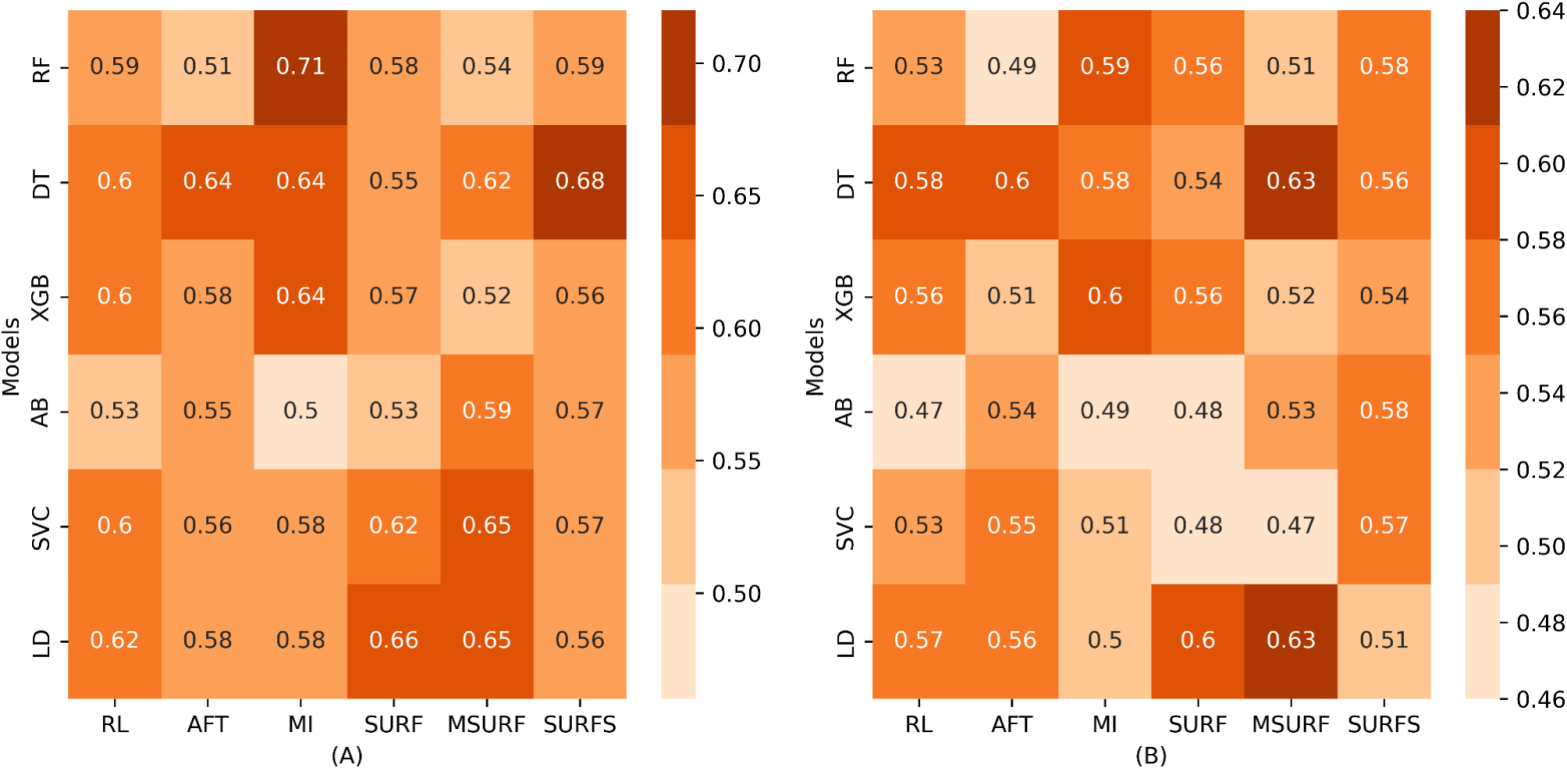
Heatmaps depicting the efficacy of various machine learning models (on the y-axis) across different feature selection techniques (on the x-axis) for predicting PFS. **(A)** AUC scores during cross-validation and **(B)** AUC scores in the external validation Phase

We explored the ability of various feature selection methods and machine learning models to build predictive models of PD-L1 expression and PFS. This evaluation was conducted using the Area Under the Curve (AUC). Six models and variable selection techniques were tested to identify the optimal combination ensuring the best performance. The AUC was utilized to assess each model with each selection technique. Figures 2 and 3 display the performance of the models (on the y-axis) and variable selection techniques (on the x-axis) for PD-L1 expression and PFS, respectively. Figure 2A shows the results obtained during the cross-validation phase, while Fig. 2B pertains to the external validation phase. The assessments of the different models and selection techniques yielded an AUC range of 0.51 to 0.76 for the training set and 0.48 to 0.66 for the validation set. These results suggest that some models struggle to distinguish between high and low PD-L1 expression classes (AUC ≤ 0.5), whereas others exhibit satisfactory predictive performances (AUC > 0.6). The Surf Star selection method provided the highest AUC of 0.66 with the support vector machine model on the training and validation set. Other models also show acceptable performances, such as GB with the Surf technique (0.64 for training and 0.63 for validation) and Gradient Boosting with the Anova technique (0.76 for training and 0.65 for validation). The lowest performance was observed with the Gradient Boosting Machine model using the Surf Star technique, with an AUC of 0.51 for training and 0.48 for validation, equating to random classification.

Figure 3A and B respectively present the model and variable selection technique performances to predict PFS, on the training and validation sets. The AUC for the training set ranges from 0.50 to 0.71, and from 0.47 to 0.63 for the validation set. The Decision Tree and Linear Discriminant Analysis models showed identical performances on the validation set (AUC = 0.63) with the Multi Surf technique. The best performances for these two models were observed with an AUC of 0.65 for Linear Discriminant Analysis and 0.62 for Decision Tree on the training set. The Adaboost model, with the ReliefF technique, displayed the lowest performance with an AUC of 0.53 during the cross-validation phase and 0.47 during the validation phase.

### Median performance of feature selection and machine learning methods

**Fig. 4:**
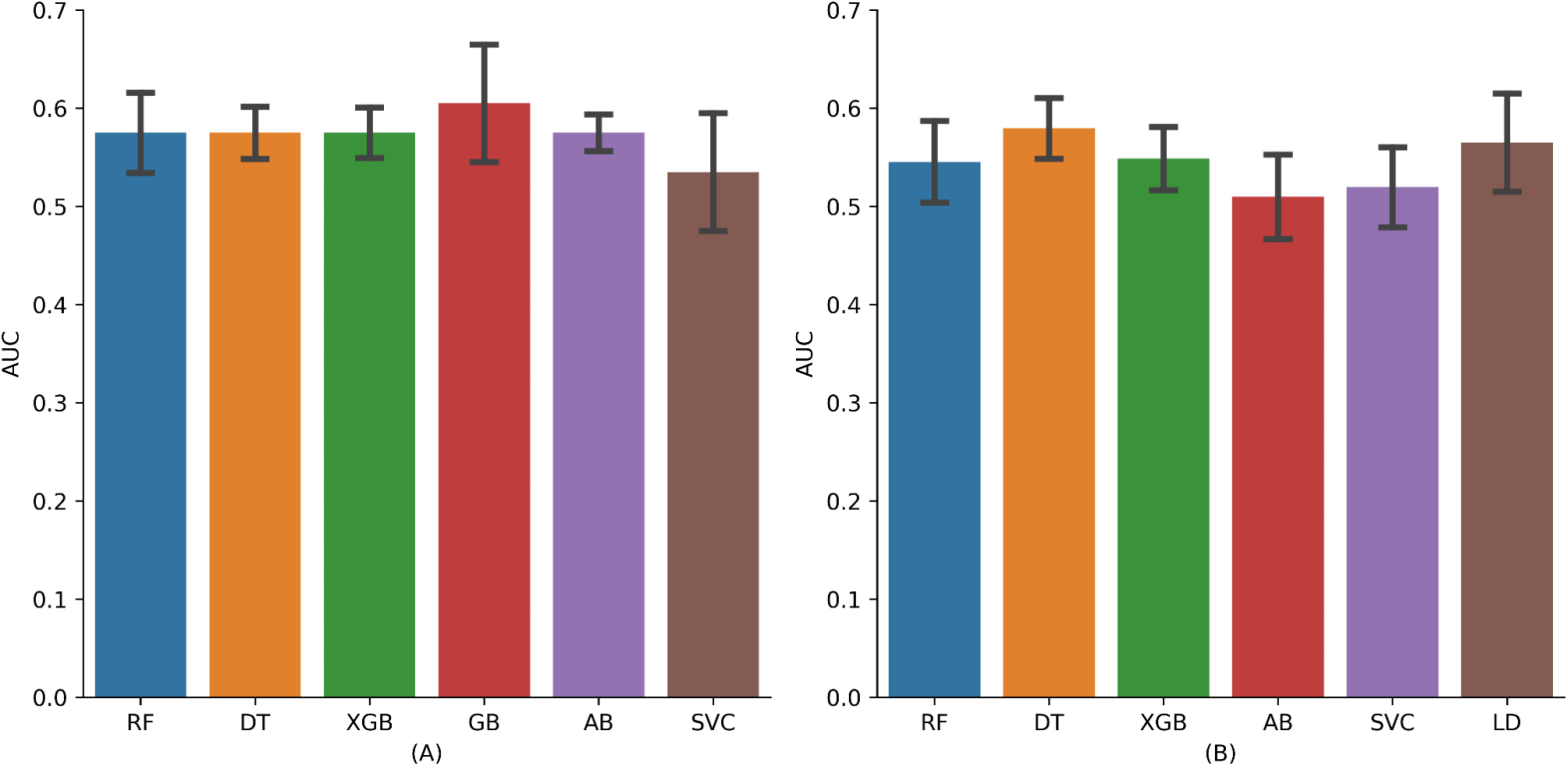
Median performance of machine learning methods to predict **(A)** PD-L1 and **(B)** PFS on the validation dataset. The color bars correspond to different machine learning models: RF (blue), DT (orange), SVC (brown), and LD (brown, only in panel B). The error bars represent the standard deviation across multiple feature selection techniques

The median performance of various models was assessed by calculating the median AUC value for each model across all feature selection techniques during the validation phase. Figure 4A depicts the median performance of PD-L1 expression prediction models. The highest median performance was recorded for Gradient Boosting (AUC: 0.61 ± 0.06, median ± standard deviation). SVC showed the lowest median performance (SVC: 0.54 ± 0.05, median ± standard deviation). Figure 4B displays the median performance of the PFS prediction models. The best performance was observed with the Decision Tree model (AUC: 0.58 ± 0.03, median ± standard deviation) and with Linear Discriminant Analysis (AUC: 0.57 ± 0.05, median ± standard deviation). Adaboost revealed the lowest median performance, with a median AUC of 0.51 and a standard deviation of 0.04. Thus, more than half of the combinations of models and variable selection techniques achieved an AUC above 0.5, distinguishing them from models yielding random results.

### Biological validation of pathomics signature

**Fig. 5:**
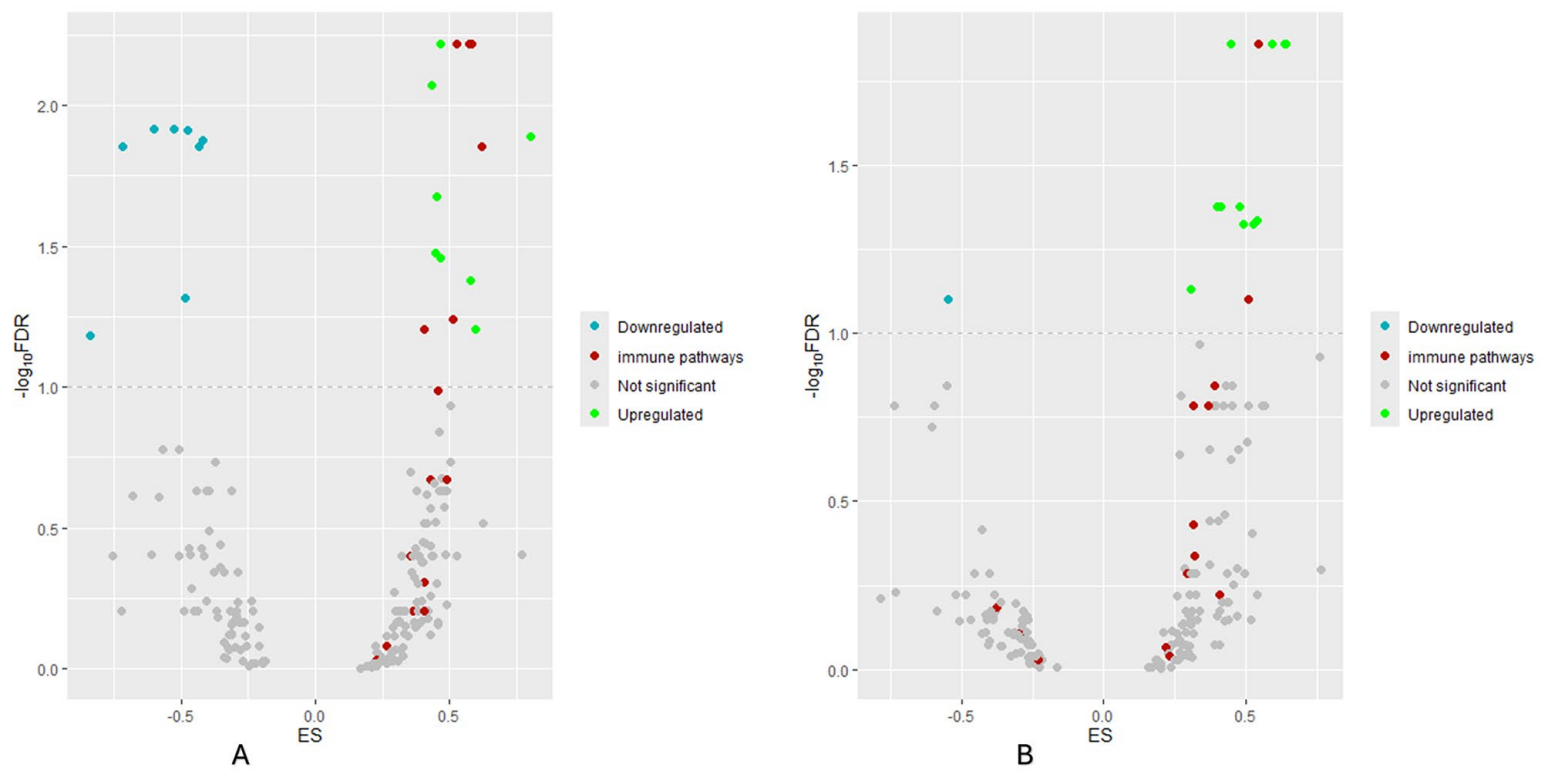
Biological validation - Volcano plot highlighting pathway enrichment analysis using **(A)** PD-L1 and **(B)** PFS. Significantly upregulated signaling pathways are highlighted in green, while significantly downregulated pathways are indicated in blue, and non-significant pathways are represented in gray. The frame lines represent the threshold of significance, corresponding to the log10FDR with a cutoff of FDR = 0.1

To explore biological features/pathways/mechanisms associated with the developed pathomics-based signatures for PD-L1 expression and PFS, we carried pathway analyses. To do so, we used the developed signature to split patients into two groups– low and high, and performed the pathway analysis. The biological validation was carried out using the best performing model from Figs. 2 and 3. Figure 5A and B illustrate the results of the GSEA analysis for PDL1 expression and PFS, showing the Enrichment Score (ES) on the x-axis and the logarithm of the False Discovery Rate (log(FDR)) on the y-axis. The GSEA results for PD-L1 revealed that 22 pathways are significantly affected with an FDR threshold below 10%: 14 are upregulated in the high PD-L1 expression group, and 8 are downregulated in the low PD-L1 expression group. Among the 14 upregulated pathways, 6 are associated with the immune pathway. Regarding the GSEA results for the model predicting PFS, 14 pathways were significant with an FDR threshold of 10%, of which 13 are upregulated and 1 is downregulated in the high group. Among these, 2 immune pathways are upregulated in the group with high PFS. We have specifically highlighted the immune-related pathways by coloring them in red. Immune pathways such as natural killer cell mediated cytotoxicity, chemokine signaling pathway, and T cell receptor signaling pathway have been identified as upregulated in patients with high levels of PDL1 and prolonged PFS. The GSEA results demonstrate the effectiveness of our biomarkers in predicting PD-L1 and PFS. In the presence of tumor cells with high PD-L1 expression, immune pathways must be activated [27–29].

Natural killer (NK) cell-mediated cytotoxicity plays a crucial role in recognizing and eliminating tumor cells. Its strong activation in the high PD-L1 group contributes to tumor cell destruction. Similarly, in the chemokine signaling pathway, leukocytes are activated, and in the T cell receptor (TCR) signaling pathway, T lymphocytes are prepared to attack tumor cells, potentially improving patient survival.

## Discussion

Arrival of ICIs in the therapeutic realm [30] have revolutionized the treatment of various cancers, especially advanced NSCLC patients. ICIs have significantly improved survival rates for patients with NSCLC treated in the first line [31]. By blocking immune checkpoints like PD-1/PD-L1, ICIs allow T cells to attack tumor cells without being deactivated. The effectiveness of ICIs is often related to the characteristics of the tumor’s immune microenvironment, with high PD-L1 expression indicating a better response to these treatments [32]. PD-L1 expression in tumors is increasingly recognized as a predictor of positive response to ICIs, and becoming the main biomarker to guide ICI treatment as shown in several clinical trials [33]. However, measuring PD-L1 expression by immunohistochemistry (IHC) is costly and requires experienced pathologists, highlighting the need to develop new biomarkers from available clinical data. These biomarkers could help distinguish patients benefiting from ICIs from those requiring other therapeutic approaches. Several studies have thus turned to OMICs data to predict response to ICIs or patient survival, although the clinical integration of these models is complex due to the need for rigorous development and validation. The importance of developing alternative biomarkers to PD-L1 has prompted researchers to explore new avenues, particularly the use of radiological images.

In this regard, various studies have focused on exploiting radiological images to develop predictive models of PD-L1, treatment response, and patient survival. Yolchuyeva et al. [34–36] analyzed several models and feature selection approaches to predict survival and PD-L1 expression in NSCLC patients treated with immunotherapy, achieving an AUC of 0.69 for PD-L1 and a C-index of 0.59 for PFS. Trebeschi et al. [37] extracted features from radiological images to create predictive models for the response to ICI treatment. Zerunian et al. [38] conducted a study on 21 patients from the same institution treated with pembrolizumab, obtaining an AUC of 0.72. Although radiological image analyses have shown some promise in predicting PD-L1 expression, treatment response, and survival, their lack of cellular and molecular resolution limits their ability to detect subtle variations in PD-L1 expression or small changes in tumor size. Despite the accuracy of these models, their interpretation remains a challenge in clinical practice. To improve clinical interpretability, some researchers have developed machine learning models using histological images to predict treatment response [6]. They used RECIST as a treatment response variable and a logistic regression model. Using the same treatment response variable, Ding et al. [7] constructed their model with pathomics features and obtained an AUC of 0.61. There are several studies in the literature that evaluated PD-L1 using deep learning methods [39–42]. A comprehensive review of AI applications in digital pathology can be found in [43]. However, studies exploring pathomics features for predicting treatment response and patient survival remain limited, and to our knowledge, none has used these features to specifically predict PD-L1. Additionally, the literature does not provide a clear understanding of which combination of feature selection strategies and machine learning algorithms yields the highest accuracy in a multi-institutional setting. A systematic comparison of various feature selection and machine learning strategies has not yet been conducted in the context of a multicentric study for NSCLC patients. Developing and validating radiomics models on larger cohorts across multiple hospitals will be crucial for advancing clinical translatability along with the biological interpretability.

With this premise, we presented a novel approach to explore alternative biomarkers for PD-L1 expression, leveraging pathomic features derived from histological images. Using data from two institutions (CHUM for model training and IUCPQ-UL for validation), we generated cell density maps and performed a detailed texture analysis by extracting Haralick features, capturing nuances in cellular structure and tumor cell arrangement. After applying a Spearman-based correlation filter to reduce redundancy, we retained 55 key variables for testing across 36 combinations of machine learning models and feature selection methods. Our models, including techniques such as Support Vector Machines and Random Forest, demonstrated strong performance, with an AUC of 0.66 for PD-L1 and 0.63 for PFS on the validation set. Biological validation through GSEA confirmed the upregulation of immune response pathways in patients with high PD-L1 expression and prolonged PFS, aligning with existing studies [27–29] and highlighting the clinical relevance of our findings. The strengths of our work include the cohorts of patients evaluated at two institutions in terms of patient diversity, data heterogeneity across centers, along with the use of two clinical endpoints. While prior studies primarily focused on deep learning models with limited translatability and interpretability without any biological validation, our approach emphasizes the extraction of interpretable morphological features that are clinically meaningful. Additionally, we validated these features with their omics counterparts, providing biological relevance for clinical integration that was lacking in earlier studies.

Despite the significant advancements presented in this study, we acknowledge its limitations. Firstly, the models were developed from a relatively modest sample size (training = 43 and validation = 25). While the sample size may not provide sufficient statistical power to generalize the findings to broader populations, the consistency of results across the training and validation datasets supports the robustness of our approach. Nevertheless, this limitation highlights the need for future studies with larger, multi-institutional cohorts to validate and expand upon our findings. Such efforts will be critical to ensuring that the predictive value of pathomic features can be reliably translated into clinical practice. Additionally, a limitation of this study is the potential variability in tissue sectioning between different pathologists. Although a standardized slide preparation and scanning protocol was used, subtle differences in tissue thickness or sectioning angles may influence image quality and subsequent analysis. Future studies should consider implementing automated tissue sectioning techniques or validating results across multiple pathologists to minimize this source of variability. Lastly, the models were trained on retrospective data. We plan to overcome these challenges in future research.

In summary, our work introduces an effective framework for predicting PD-L1 expression and PFS in patients with NSCLC treated with immunotherapy. Its robustness during multi-site external validation, along with biological validation that aligns with current knowledge, attests to its reliability. Our pathomics approach offers a complementary perspective to the existing immunohistochemistry (IHC)-based PD-L1 testing, addressing key limitations of current diagnostic workflows. While IHC is a well-established standard, it is associated with significant costs, requires specialized pathologists, and is subject to inter-observer variability. In contrast, our method leverages readily available H&E-stained slides, eliminating the need for additional molecular tests or costly reagents. This makes the approach particularly advantageous in resource-limited settings where access to advanced diagnostic facilities may be constrained. Moreover, the interpretability of our machine learning models enhances their clinical utility. By using handcrafted features derived from histological images, we bridge the gap between computational analysis and biological insight. These features, biologically validated and linked to immune pathways, provide clinicians with a tool to stratify patients based on their likelihood of responding to immunotherapy, independent of PD-L1 expression levels measured by IHC. Importantly, this approach preserves tissue integrity, as it does not rely on destructive molecular analyses, making it suitable for scenarios where limited tissue samples are available. By providing a cost-effective and accessible alternative, our pathomics-based models could be integrated into routine clinical workflows with further validation, thus, complementing PD-L1 IHC testing and broadening the scope of precision oncology.

## Data Availability

Data presented in this study are not publicly available at this time but may be obtained from the corresponding author, Venkata Manem upon reasonable request.
